# Is cirrhotic cardiomyopathy a risk factor for post-reperfusion syndrome during liver transplantation?

**DOI:** 10.1186/cc13394

**Published:** 2014-03-17

**Authors:** E Scarlatescu, G Droc, D Tomescu

**Affiliations:** 1 Fundeni Clinical Institute, Bucharest, Romania

## Introduction

A period of hemodynamic instability following revascularization of the liver graft during liver transplantation is frequently observed and is termed post-reperfusion syndrome. Recent studies showed the existence of a specific heart disease associated with cirrhosis termed cirrhotic cardiomyopathy (CCM). The aim of this study was to investigate whether the CCM has an influence on the development or the severity of post-reperfusion syndrome.

## Methods

Fifty-two consecutive liver transplant patients were included in a retrospective observational study. The variables recorded were: age, etiology of the liver disease, MELD and MELD Na scores, the associated pathologies, the length of the QT interval, and plasma levels of brain natriuretic peptide (BNP). The patients with known renal or heart disease and the recipients of organs from extended criteria donors were excluded from the study. The QT interval was corrected for the heart rate using Bazett's formula (QTc). Statistical analysis was performed using SPSS Statistics v.19.1.

## Results

In our study the criteria used to define post-reperfusion syndrome relied on the hemodynamic changes that occurred at reperfusion. Preoperative echocardiography showed normal systolic and diastolic function at rest in all of the patients. For the identification of patients at risk for CCM we used two of the supportive criteria from the recent definition of CCM: prolonged QTc interval and increased BNP levels. The study group included 28 men (53.8%) and 24 women. Mean (± SD) age was 50.5 (± 11.4). Mean MELD and MELD Na scores were respectively 15.51 (± 5.43) and 18.9(± 6.22). The value of BNP correlated well with the length of the QTc interval (*P *= 0.005), and with MELD and MELD Na scores (*P *= 0.025 and *P *= 0.001). In our study, post-reperfusion syndrome occurred in 63.4% of the patients. We could not find a correlation between post-reperfusion syndrome and the BNP levels (*P *= 0.85) or the prolonged qTc interval (*P *= 0.38). The post-reperfusion syndrome did not correlate with the severity of the liver disease as assessed by MELD and MELD Na scores. The severity of post-reperfusion syndrome did not correlate with QTc prolongation or BNP levels.

## Conclusion

Reperfusion is a critical time during liver transplantation. The clinical predictors of post-reperfusion syndrome are still under debate [1]. Our study showed that the post-reperfusion syndrome is not correlated with the severity of the liver disease or with the presence of risk factors indicating CCM.
